# Intraocular route of AAV2 vector administration defines humoral immune response and therapeutic potential

**Published:** 2008-09-24

**Authors:** Qiuhong Li, Rehae Miller, Ping-Yang Han, Jijing Pang, Astra Dinculescu, Vince Chiodo, William W. Hauswirth

**Affiliations:** Department of Ophthalmology, University of Florida, Gainesville, FL

## Abstract

**Purpose:**

Safety and efficiency are critical for successful gene therapy. Adeno-associated viral (AAV) vectors are commonly used for gene transfer in both human and animal studies. However, administration of AAV vectors can lead to development of neutralizing antibodies against the vector capsid, thus decreasing the efficiency of therapeutic gene transfer and preventing effective vector readministration. We investigated immune responses to different routes of ocular administration and readministration of AAV vectors, and the effect of previous exposure of AAV vector in one eye on the transduction efficacy of subsequent intraocular AAV-mediated gene delivery to the partner eye.

**Methods:**

We tested two vector systems. One contained a cDNA encoding a secreted pigment epithelial derived factor (PEDF) cDNA under the control of a Cytomegalovirus (CMV) enhancer and chicken β-actin promoter (CBA; AAV2-CBA-PEDF) and was tested in a murine model of laser-induced choroidal neovascularization (CNV). The other vector contained a cDNA encoding the intracellular reporter green fluorescent protein (GFP) under the control of the same promoter (AAV2-CBA-GFP). Animals were divided into groups and received sequential injections at different combinations of either intravitreal or subretinal routes. CNV was evaluated by fluorescein angiographic choroidal flat-mount image analysis. The expression of GFP was analyzed in retinal sections by direct fluorescence imaging. Antibodies against AAV2 capsid and transgenes were analyzed by ELISA using serum samples collected before injection and different time points after the injection. Neutralizing antibodies were characterized by in vitro assays.

**Results:**

Various ocular compartments responded to AAV administration differently. Intravitreal administration of AAV vectors, which resulted in transduction of inner retina (primarily retinal ganglion cells), generated a humoral immune response against AAV capsid that blocked vector expression upon readministration via the same route into the partner eye. In contrast, it had no effect on vector readministered into the subretinal space of the partner eye. Additionally, subretinal administration of vector did not trigger any humoral immune response against AAV capsid, and had no effect on subsequent administration of vector either intravitreally or subretinally into the partner eye.

**Conclusions:**

These findings have important clinical implications for the design of AAV-mediated ocular gene transfer for retinal diseases, particularly if both eyes require sequential treatment.

## Introduction

Despite the many advantageous properties of adeno-associated viral (AAV) vectors to deliver potentially therapeutic genes to the tissue of choice, preexisting immunity due to prior exposure with wild-type (wt) AAV vectors in the majority of the human population could potentially limit their therapeutic usefulness [1-6]. In animal studies, preimmunization with recombinant AAV vectors has resulted in reduction or lack of transgene expression [3,7,8] and correlated with the presence of neutralizing antibody (nAB) found in the serum. Moreover, studies of repeated administration of AAV vectors indicate that immune responses generated after an initial administration may prevent or mute further vector-mediated cell transduction [9-14]. The presence of high levels of nAB against wt AAV also reduced AAV-mediated gene transfer in the brain [4]. Several strategies have been developed to circumvent these responses (reviewed in [15]).

The eye is considered to be an immunologically protected space (reviewed in [16]). The origin of this immune privilege is complex and is generated by multiple layers and mechanisms including the blood-retina barrier and other physical barriers, an immunosuppressive microenvironment, and the existence of deviant systemic immunity that limits the production of proinflammatory effector cells (reviewed in [17]). These mechanisms provide the eye with a degree of immune protection that lacks acute, destructive inflammation, thus sparing the delicate visual axis which is incapable of regeneration after early development. It is commonly assumed that preexposure to AAV may not pose significant problems with regard to the performance of AAV vectors in the eye because of this ocular immune privilege. Few studies have focused on the impact of previous systemic immune response to AAV on transduction efficacy of AAV vectors in distinct ocular spaces, such as the intravitreal cavity and subretinal space. In addition, how the immune system responds to administration and readministration of AAV vectors in these ocular compartments is poorly understood.

In this study, we investigated immune responses to different routes of ocular administration and readministration of AAV vectors, and the effect of previous exposure of AAV vector in one eye on the transduction efficacy of subsequent intraocular AAV-mediated gene delivery to the partner eye. We tested two vector systems. One contains a cDNA encoding a secreted pigment epithelial derived factor (PEDF) under the control of a CMV enhancer and chicken β-actin promoter (CBA; AAV2-CBA-PEDF) which has been previously shown to inhibit the choroidal neovascularization (CNV) in a murine model [18]. The other vector contains a cDNA encoding the intracellular reporter green fluorescent protein (GFP) under the control of the same promoter (AAV2-CBA-GFP). We show that, although the eye is an immune-privileged site, various ocular compartments respond to vector administration differently. Intravitreal administration of AAV vectors, which results in transduction of inner retinal cells, generated a humoral immune response against AAV capsid that blocked expression from readministered intravitreal vector in the partner eye. However, intravitreal vector in the first eye had no effect on readministering vector into the subretinal space of the partner eye where photoreceptor and retinal pigment epithelial (RPE) cells were transduced. Moreover, if the initially treated eye received subretinal vector, no humoral immune response against AAV capsid was elicited, and no effect on subsequent administration of the AAV vector either intravitreally or subretinally ensued. Although both intramuscular and intravitreous injections elicited production of neutralizing antibody against AAV2 capsid, the profile of immunoglobulin classes was different, reflecting the ocular regulatory immune modulation of the immune response.

## Methods

### AAV serotype 2 production and purification

A fused CBA was used to drive expression of GFP and human PEDF in recombinant AAV2, based on pTR-UF vectors [19]. The construction and production of CBA-PEDF-AAV2 has been described previously in which the human PEDF gene is expressed from the CBA promoter [18]. Vectors were produced and purified as previously described [20,21]. Briefly, HEK 293 cells were cotransfected with the appropriate pTR-UF and the helper plasmid pDG DNAs for 48–60 h. Cells were harvested, and the crude lysate purified through an iodixanol step gradient followed by Mono-Q FPLC chromatography. The vector genome (vg) titers of AAV2 particles were determined by real-time PCR.

### Animals, injections, and tissue process

All mouse experimentation was performed under approved protocols from the University of Florida Animal Care and Use Committee and in accordance with National Institutes of Health guidelines. Wild-type C57BL/6J mice (6-8 weeks of age) were obtained from The Jackson Laboratory (Bar Harbor, ME). For intramuscular (IM) injections, approximately 4x10^10^ vector genome (vg) per animal was delivered (n=5). For ocular injections, 1 μl of AAV2-CBA-PEDF vector (approximately 4x10^9^ vg) was injected intravitreally (15 mice per treatment group) or 1 μl of AAV2-CBA-GFP vector (approximately 2x10^9^ vg) was injected either intravitreally or subretinally (5 animals per group). Detailed techniques for intravitreal and subretinal injections have been described previously [18,22]. For GFP expression analysis, mice were sacrificed at the end of the experiments. The mice were sacrificed by ketamine (150 mg/kg)/xylazine (10 mg/kg) overdose followed by cervical dislocalization. Their eyes were enucleated, fixed in 4% paraformaldehyde for 2–4 h at room temperature, cryoprotected in 30% sucrose, embedded in optimal cutting temperature (OCT) embedding medium, and quickly frozen. Transverse sections of the eye were cut at 12 μm, mounted on slides, and GFP fluorescence was examined under a Zeiss microscope (Axiovert 200, Carl Zeiss MicroImaging, Inc., Thornwood, NY) equipped with epifluorescence illumination and a digital camera (AxionCamMR, Carl Zeiss MicroImaging, Inc., Thornwood, NY).

### Laser-induced CNV mouse model

The mouse CNV model was induced as previously described [18]. Briefly, adult mice were anesthetized, and eyes dilated. A 532 nm diode laser (Oculight SLx; Iridex Co., Mountain View, CA) was used with a 100 μm spot size at 0.1 s exposure and 300 mW power. Laser photocoagulation was delivered through a slit lamp with a cover slide employed as a contact lens. A pattern of 4–6 lesions was placed concentrically around the optic nerve. Formation of a bubble indicated rupture of Bruch’s membrane. To quantitatively evaluate CNV two weeks following laser treatment, we prepared flatmounts of sclera, choroid, and RPE that we imaged using a Zeiss fluorescence microscope (AxioVision, Carl Zeiss MicroImaging, Inc., NY). The neovascular area at each laser lesion was measured with a Zeiss AxioVision Software measurement tool, and the data was expressed in μm^2^.

### Enzyme-linked immunosorbent assays

To detect serum antibodies to AAV2 capsid, we coated enhanced protein-binding ELISA plates with 10^9^ vg/ml of AAV2 at 4 °C overnight. The plates were blocked at 37 °C for 2 h then incubated at 4 °C overnight with serially diluted anti-AAV2 monoclonal antibody (Industries International, Concord, MA) or 1:50, 1:100, 1:200, or 1:400 dilutions of mouse sera. Next, the plates were incubated with HRP-conjugated anti-mouse Ig at 37 °C for 2 h, then with TMP substrate and H_2_O_2_. The reaction was stopped by H_3_PO_4_ and read at 450 nm on an EL808 plate reader. The titer of anti-AAV2 antibodies was calculated based on the standard curve of the commercial antibody determined in parallel. Each value was determined in triplicate.

To detect antibodies against transgenes (GFP and PEDF), we coated the microplates with either 5 μg/ml purified GFP (generously provided by Clay Smith, University of Florida), or 5 μg/ml purified PEDF (BioProduct MD, LLC., Middletown, MD), followed by incubation with mouse sera as described in the previous section. Commercial monoclonal antibodies to GFP (Invitrogen, Carlsbad, CA) and PEDF (Santa Cruz Biotechnology Inc., Santa Cruz, CA) were used as positive controls. Isotyping of the antibody responses was performed using a commercial kit (Southern Biotechnology, Birmingham, AL) according to the manufacturer’s instruction.

### Neutralizing antibody assay

To detect neutralizing antibodies to AAV2, we incubated 1:20, 1:60, 1:180, 1:540, 1:1620, or 1:4860 mouse serum samples with 10^8^ vg AAV2-GFP in 25 µl of PBS for 2 h at 4 °C. This mix was added to each well of HEK 293 cells grown in a 24 well plates (to achieve a multiple of infection (MOI) at 1000). The cells were grown at 37 °C in 5% CO_2_, in Dulbecco's Modified Eagle's Medium (DMEM; HyClone, Logan, UT) containing 5% FBS (HyClone). Each sample was run three times. GFP expression was evaluated 48 h after infection by cell counting. Percentage of inhibition was calculated with no-antibody control samples as a reference.

### Statistics

All values are expressed as means±SD. Statistical analysis was performed with unpaired, two-tailed Student t-tests for single comparisons. A p-value of < 0.05 was taken to indicate a significant difference.

## Results

### Intravitreal injection of CBA-PEDF-AAV2 results in a humoral immune response against AAV2 capsid and prevents therapy upon vector re-administration in the contralateral eye

In an initial attempt to emulate clinical situations where contralateral eyes are treated sequentially with therapeutic genes delivered via AAV vectors, we tested the CBA-PEDF-AAV2 vector in a laser-induced mouse CNV model. We chose this construct and animal model because previous results have shown that it efficiently inhibited neovascularization in laser-induced CNV mouse model [18].

Wild-type C57BL/6J mice were divided into three groups. Group 1 received a single intravitreal injection of CBA-PEDF-AAV2 vector in right eye only. Group 2 received an intravitreal injection of the vector into the right eye; two months later, they were given a second intravitreal injection of the vector in the left eye. Group 3 received an IM injection of the vector first to preimmunize systemically with the vector. They were then given an intravitreal injection of vector into the right eye. Both eyes from all groups were then lasered to induce CNV two months after the second injection or at an equivalent time for Group 1. All mice were sacrificed two weeks after laser treatment to quantitatively evaluate the level of CNV development.

**Figure 1:**
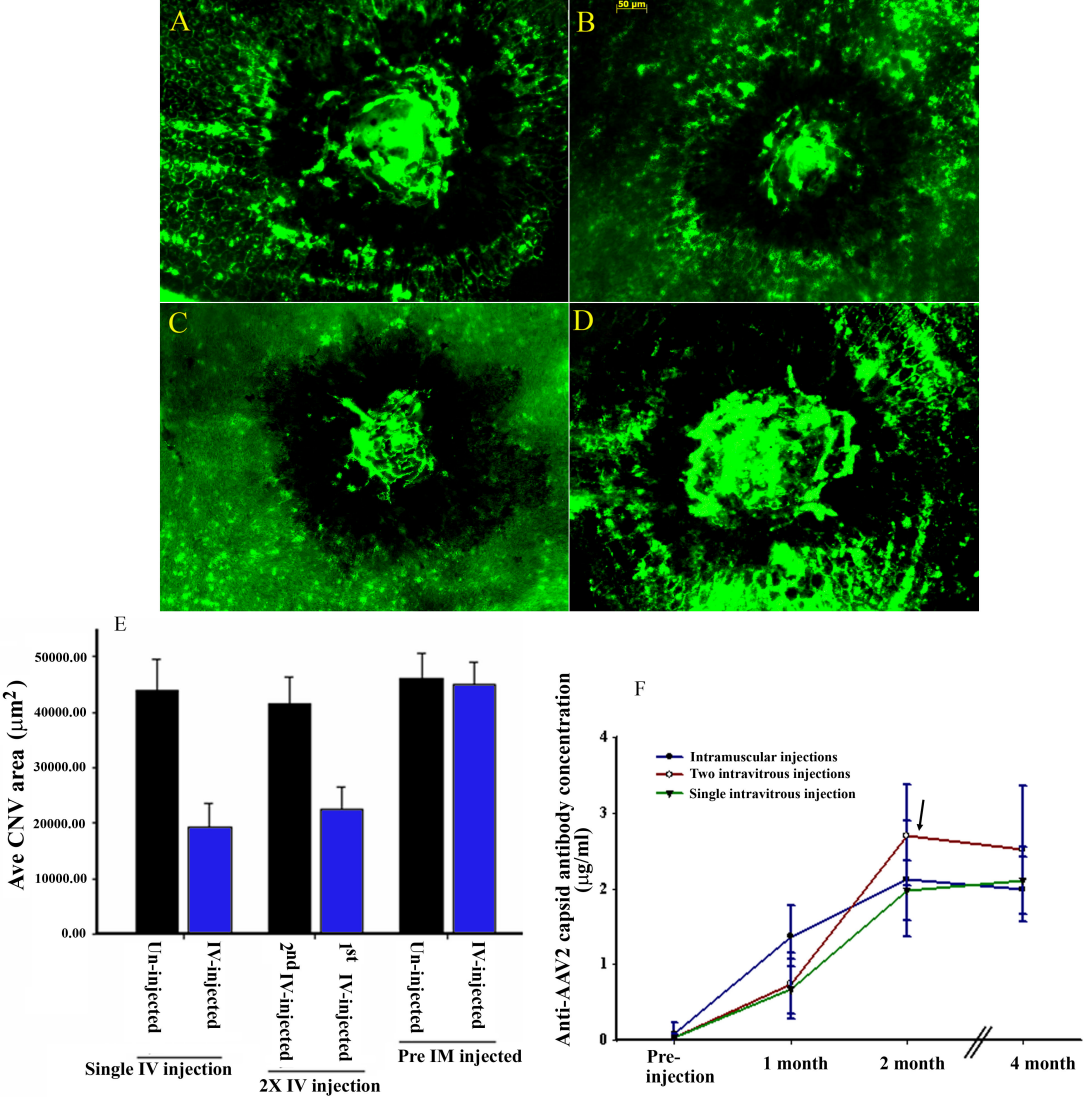
Therapeutic effect of CBA-PEDF-AAV2 on choroidal neovascularization development and antibody responses to AAV2 capsid following single and sequential intravitreal injections. **A**-**D**: Representative CNV images from mouse eyes that were untreated (**A**), received a single intravitreal (IV) treatment (**B**), received first IV treatment (**C**), and received a second IV treatment (**D**) of AAV2-PEDF. **E:** The therapeutic effect of CBA-PEDF-AAV2 on CNV development was evaluated in animals that received single IV ocular injections in naïve and preimmunized mice (5 animals each type of injection), and two sequential IV injections (15 animals were used). The CNV area was averaged from 25 laser lesions (from 5 mice) for mice that received a single IV injection and pre-intramuscular (pre-IM) administration of the vector, and 75 laser lesions (from 15 mice) for mice that received two sequential intravitreal injections (2X-IV). **F**: Antibody response to AAV2 capsid in animals received a single intramuscular injection, intravitreal, and two sequential intravitreal injections overtime. Five animals for each type vector injection were used. Arrow indicates the second injection time.

There was a significant reduction of CNV (Figure 1B,E), as reported previously [18], in eyes from mice that received a single intravitreal injection of AAV2-CBA-PEDF (Group 1). Right eyes that received intravitreal injection of the vector followed by an intravitreal vector injection into their left eyes (Group 2) showed a similar level of CNV reduction as mice receiving a single treatment (Figure 1C,E). However a second vector administered intravitreally to contralateral eyes had no effect on CNV development (Figure 1D,E). Vector-treated eyes from mice previously exposed to AAV vector through IM injection also showed no therapeutic difference from untreated eyes (Figure 1E). Thus it appeared that the mouse’s immune status with regard to AAV vector may define whether or not intravitreal vector could effectively express a therapeutic gene.

To test this idea, the anti-AAV capsid immune status of these animals was determined from serum samples collected before the first injection, at one month, and two months after the first injection (before second injection), then at four months after the first injection. The presence of anti-AAV2 capsid antibodies in the serum samples was analyzed by ELISA. Serum antibody against AAV2 capsid was found in all animals receiving intravitreal injections at a level equivalent to that seen in animals receiving IM administration, Group 3 (Figure 1F). No CNV therapy was evident in Group 3 animals previously immunized with IM vector. Therefore the presence of circulating capsid antibody correlated with the failure of intravitreal AAV2-CBA-PEDF vector to inhibit CNV.

### The route of intraocular vector delivery defines systemic immune responses against AAV2 capsid and affects transgene expression from readministered vector in the partner eye

The inability of vector-treated second eyes to respond to the antineovascular effects of AAV2-CBA-PEDF after laser treatment prompted us to examine in more detail both the immune status and therapeutic potential of ocular vector as a function of its intraocular site of delivery. Ocular tissues, particularly the anterior chamber, vitreal cavity, and subretinal space, are immune privileged sites (reviewed in [16]). It is generally assumed that neutralizing antibodies in the circulation cannot penetrate the blood-ocular-barriers; thus previous exposure to AAV, which may diminish or completely prevent systemic administration of AAV vectors, should have minimal effect on AAV-mediated gene transfer in ocular tissues. The aforementioned view of the eye and the immune system prompted us to systematically investigate how the immune system responds to AAV-mediated gene transfer into different intraocular compartments and the impact of such responses upon readministering vector to the contralateral eye of the same animal.

For these experiments, we used a GFP reporter gene (under the control of the same CBA promoter in the same AAV2 vector backbone as described), so that vector transduction of different retinal cell types could be easily visualized in tissue sections. Wild-type C57 mice were divided into four groups and subjected to different combinations of sequential ocular injections. Mice received either an intravitreal (IV) or subretinal (SR) injection into one eye, then a second invitreal or subretinal injection two months after the first. All mice were sacrificed one month after second injection (three months after the first injection). Group 1 mice received vector intravitreally followed by intravitreal vector in the partner eye two month after the first vector administration, Group 2 received vector intravitreally followed by subretinal vector in the partner eye, Group 3 received vector subretinally followed by intravitreal vector in the partner eye, and Group 4 received vector subretinally followed by subretinal vector in the partner eye. Retinas were then analyzed in transverse tissue sections for GFP expression. Blood samples were collected before vector injection, and then at one month, two months (just before the second injection), and finally at three months after the first injection. These samples were analyzed for the presence of antibody against AAV2 capsid. We chose to perform the second injection at two months after the first, because in previous experiments an intravitreal delivered vector elicited serum anti-AAV antibody levels that peaked at approximately this time (Figure 1F).

**Figure 2:**
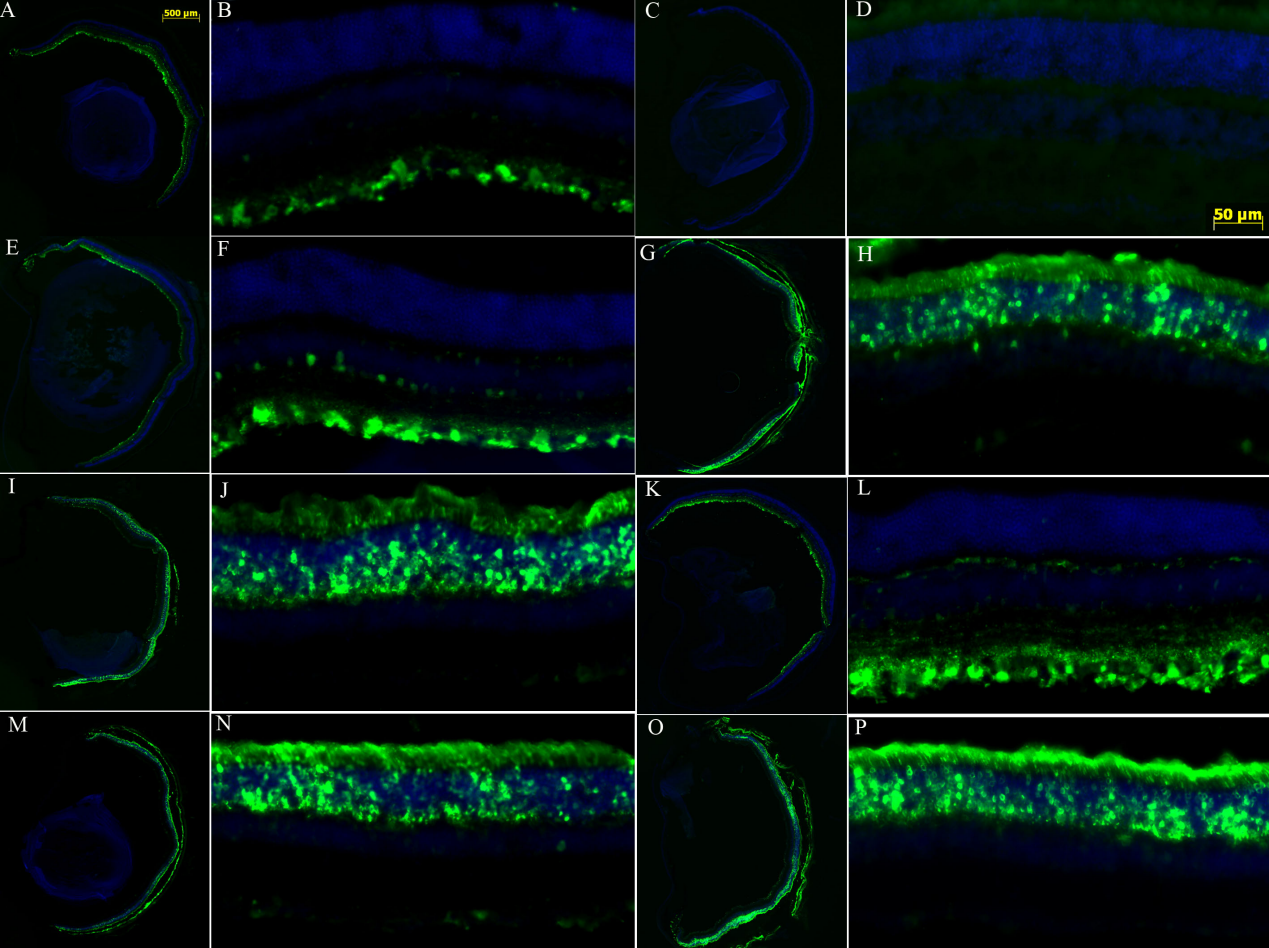
GFP expression in retinas receiving different routes of intraocular vector sequentially in partner eyes. Representative images of retinas from each treatment group listed in Table 1 are shown in the same order. **A, B, E, F, I, J, M,** and **N** show retinas that received first eye vector injections. **C, D, G, H, K, L, O,** and **P** present retinas that received second eye vector injections. **A-D** show retinas that received two sequential intravitreal injections (IV-IV) vector injections. **E-H** are from retinas that were given two sequential injections with first eye injected intravitreally and second subretinally (IV-SR) vector injections. **I-L** present retinas that received two sequential injections with first eye injected subretinally and second intravitreally (SR-IV) vector injections. **M-P** represent retinas that received two sequential subretinal injections (SR-SR) vector injections. A low-magnification image from each eye is shown on the right for each treatment group with a higher magnification image from the same eye next to it on the right (e.g., image **B** is taken from **A**, image **D** is taken from **C**, and so forth).

Results of these experiments are summarized in Table 1. Images of GFP expression in representative retinas from both eyes in each group of animals are shown in Figure 2. In Group 1 the initial intravitreal injection of CBA-GFP-AAV2 efficiently transduced mainly retinal ganglion cells as expected (Figure 2A,B,E,F). In contrast, a second intravitreal injection two months later into the partner eye yielded no transduction of any retinal cells (Figure 2C,D). Yet when the second vector injection into the partner eye was delivered to the subretinal space (Group 2), there was the expected efficient GFP transgene expression in photoreceptors and RPE cells (Figure 2G,H). Conversely, an initial subretinal vector prevented neither subsequent intravitreal (Group 3, Figure 2I,J,K,L) or subsequent subretinal (Group 4, Figure 2M,N,O,P) vector from transducing target retinal cells normally.

**Table 1:** Summary of retinal GFP expression in animals receiving different combinations of sequential intraocular injections of AAV2-CBA-GFP vector

**Group**	**First injection**		**Second injection**	
	**GFP expression**	**Cell types**	**GFP expression**	**Cell types**
Group 1 (IV-IV)	+++	mainly RGC	none	n/a
Group 2 (IV-SR)	+++	mainly RGC	+++	PR and RPE
Group 3 (SR-IV)	+++	PR and RPE	+++	mainly RGC
Group 4 (SR-SR)	+++	PR and RPE	+++	PR and RPE

Group 1 animals received two sequential intravitreal (IV) injections of the vector. Group 2 animals received IV injection in the first eye, followed by SR injection in the second eye. Group 3 received SR injection in first eye, followed by IV injection in the second eye. Group 4 animals received two sequential subretinal (SR) injections. Abbreviations: intravitreal (IV); subretinal (SR); retinal ganglion cells (RGC); photoreceptors (PR); retinal pigment epithelial cells (RPE), green fluorescent protein (GFP).

**Figure 3:**
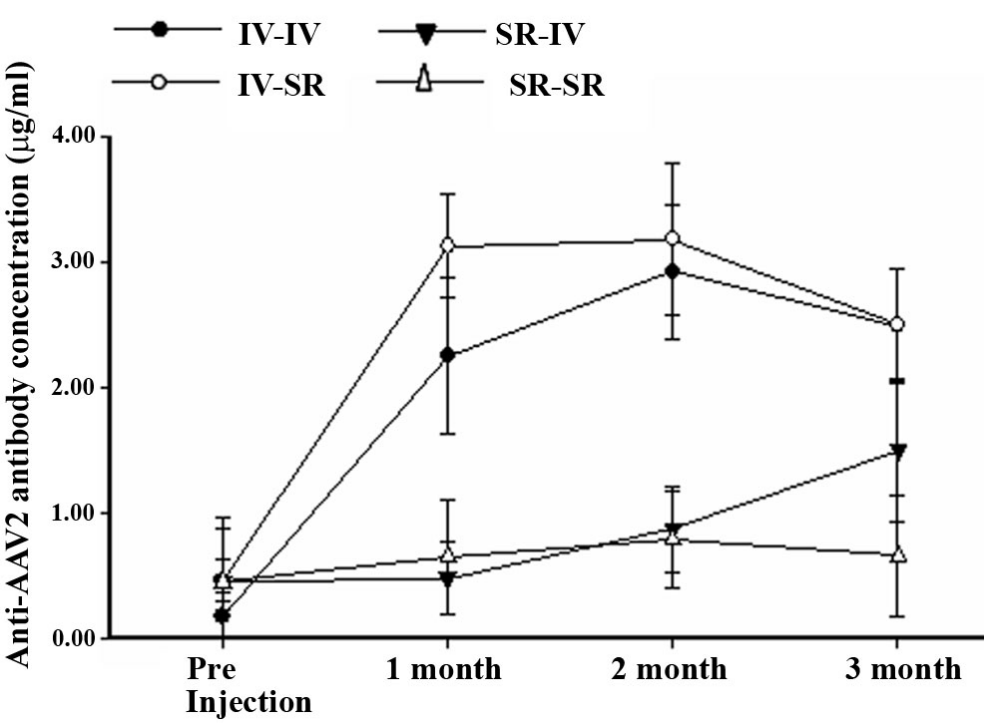
Serum antibody responses to AAV capsid in animals after different sets of sequential intraocular AAV2-CBA-GFP injection. Five animals for each group were used. Group 1 animals received two sequential intravitreal (IV) injections of the vector. Group 2 animals received two sequential subretinal (SR) injections. Group 3 received SR injection in first eye, followed by IV injection in the second eye. Group 4 animals received IV injection in the first eye, followed by SR injection in the second eye.

The absence of GFP expression in the second eyes of Group 1 mice after an initial intravitreal vector exposure correlated with the presence of antibody against AAV2 after this first vector (Figure 3). This was also seen in the initial experiment with intravitreal CBA-PEDF-AAV2 described (Figure 1E). The presence of serum anti-AAV2 antibody in Group 2, which received an intravitreal injection first and then a subretinal injection of the vector, did not prevent photoreceptor and RPE cell transduction by the second vector (Figure 2G,H). Mice that received subretinal injections first did not generate significant levels of anti-AAV2 antibody (Figure 3); such vector had no effect on GFP expression from subsequently readministered vector into the partner eye, either intravitreally or subretinally (Figure 2I-P, and Table 1).

### Identification of neutralizing antibodies to AAV2 capsid

**Figure 4:**
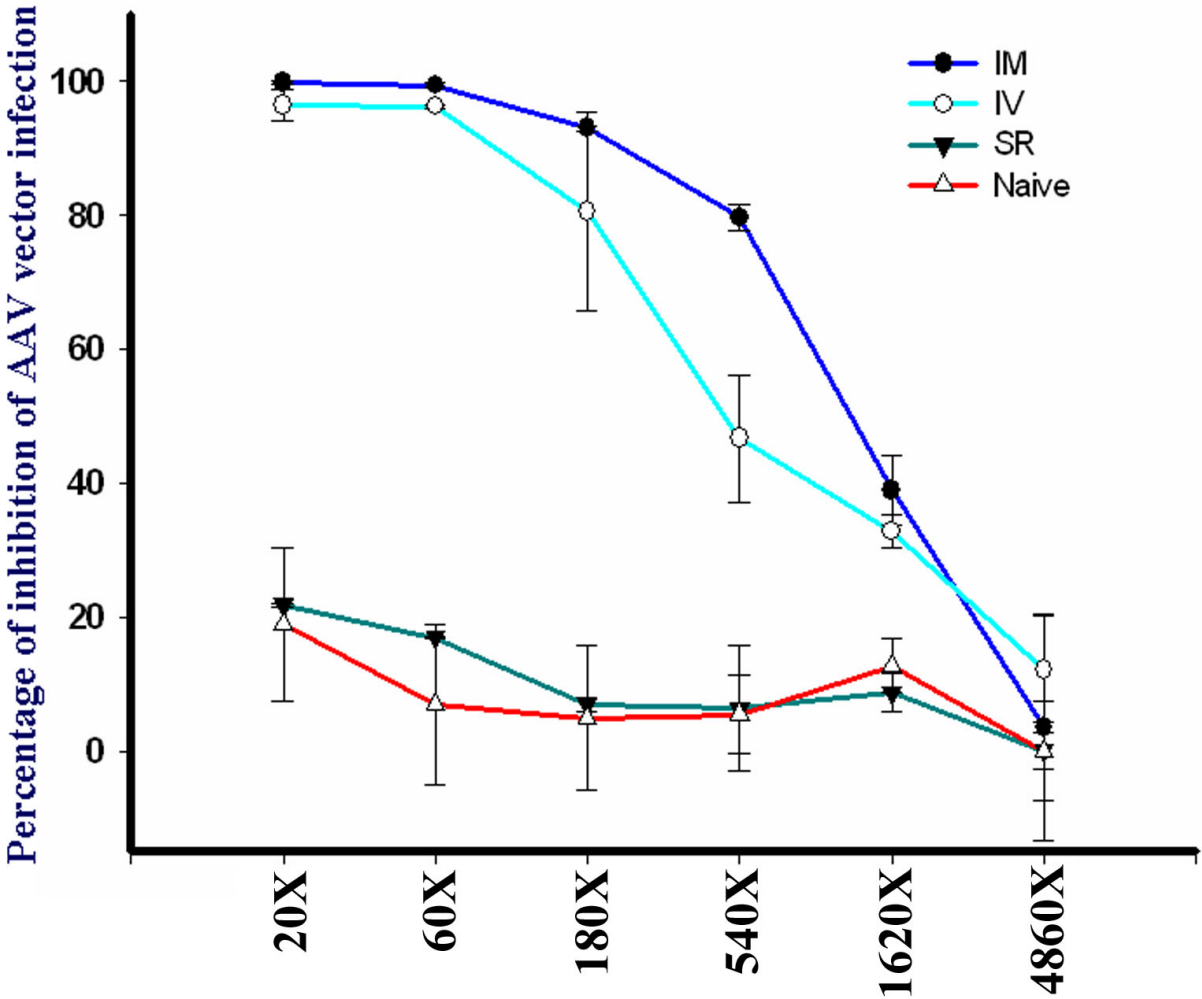
Neutralizing antibodies to AAV2 capid using in vitro assay. Serum samples were collected from animals at four weeks after receiving different route of injections (IM: intramuscular; IV: intravitreal; SR: subretinal), as well as from naïve animals that have not been exposed to AAV vector previously. Each sample was serially diluted. Each serum sample from serial dilutions was incubated with AAV GFP virus before infecting HEK293 cells. The percentage of inhibition was calculated using no-serum AAV2-GFP as the reference. Abbreviations: intramuscular injection (IM); intravitreal injection (IV); subretinal injection (SR). Serum samples from 4 animals were used for each route of injection of the AAV vector.

To determine the neutralizing capability of antibodies against AAV2 capsid from mice who received different injections of AAV2 (IV, SR, and IM), we incubated serum samples with threefold serial dilutions with CBA-GFP-AAV2 vector (MOI 1000) for 2 h at 4 °C before infecting HEK293 cells. As shown in Figure 4, serum samples from mice that received IV and IM injection of AAV2 (four weeks postinjection) contained neutralizing antibodies to AAV2 capsid, whereas serum from mice received SR injection did not show significant level of nAb to AAV2 capsid.

We also tested humoral responses to transgenes by ELISA. No antibodies against either the cytoplasmic protein GFP, or the secreted protein PEDF were detected in mouse sera received either intramuscular or intravitreal injections (data not shown).

To compare the immunoglobulin profile, we also performed isotyping using commercial kit. Although mouse sera from both IM and IV injections contained neutralizing antibodies against AAV2 capsid (Figure 3 and Figure 4), the IgG subclasses are very different: sera from mice that received IM injections contained high level of IgG2a, but scant or no detectable levels of IgG2b, whereas sera from mice that received IV injections contained mostly IgG2b and no detectable IgG2a.

## Discussion

AAV vectors have many attractive features for safe and efficient gene therapy, including their lack of pathogenesis, low toxicity, ability to efficiently infect both dividing and nondividing cells in a broad range of host tissues or organs, and long-term gene expression [23,24]. The eye, considered an extension of the CNS, has many unique advantages as a target for gene therapy to treat both inherited and acquired ocular diseases. The ability of AAV vectors to efficiently transduce target retinal cell types has been exploited to successfully transfer therapeutic genes into photoreceptors, retinal pigment epithelium, and the inner retina to treat a variety of retinal diseases causing blindness [25-29] [22,30,31].

An estimated 80% of the population maintain antibodies to the capsid proteins of wt AAV2, and 30%–84% demonstrate the presence of nAb [2,32-37]. Since natural exposure to wt AAV is quite common in human population, this poses a possible threat to the efficacy as well as the safety of AAV vector administration [1-6]. Moreover, studies of repeated administration of AAV vectors into nonocular tissue indicate that immune responses generated after the first administration may prevent further application [9-14]. It is generally assumed that previous exposure to AAV will not pose significant problems for the efficacy of AAV vectors in the retina, an immune-privileged site. This notion is supported by the observation that subretinal readministration of AAV vectors resulted in additional transduction events despite the presence of serum antibodies to AAV vectors [38,39]. However, few studies have examined the impact of previous systemic immune response to AAV capsid on transduction and therapeutic efficacy of AAV vectors in different ocular tissues, and little is known about how the immune system responds to AAV vector administration and readministration into different compartments of ocular tissues.

In the studies described in this report, we investigated immune responses to different routes of ocular administering and readministering AAV vectors, and the effect of previous exposure to AAV vector on the therapeutic efficacy of subsequent intraocular AAV-mediated gene delivery. We tested two vector systems, CBA-PEDF-AAV2 in a murine laser induced CNV model, and CBA-GFP-AAV2, where the ocular tissue transduction can be directly visualized. We showed that, although both vitreal cavity and subretinal space are immune-privileged sites [40-47], when AAV vectors were delivered into these distinct ocular compartments, they triggered immune responses differently. Intravitreal administration of AAV vectors, which mostly results in transduction of inner retina (mainly retinal ganglion cells), generated humoral immune response against AAV capsid to a level equivalent to systemic (IM) administration of the vector. The presence of neutralizing antibody completely blocked expression from readministered vector via the same route, but had no effect on readministered vector if directed into the subretinal space where photoreceptor and RPE cells are transduced. Initial subretinal administration of AAV vector did not trigger any humoral immune response against AAV capsid and had no effect on subsequent intravitreal or subretinal administration of vector.

Antigens introduced in the ocular microenvironment are known to induce immune deviation. Such deviation has the characteristics of antigen-specific suppression of classical Th1 immune responses such as delayed-type hypersensitivity (DTH) and inability of producing complement-fixing antibodies, thus lacking classic inflammatory responses seen in other nonimmune-privileged tissues. The best characterized ocular immune deviation is known as anterior chamber-associated immune deviation (ACAID) [48-50]. It is now known that ACAID is a complex immunoregulatory phenomenon that involves multiple organ systems and cell populations. Such immune privilege has also been extended to the vitreous cavity (VC) [41,45,46,51,52], and subretinal (SR) space [41,43,44,47,53; also see recent review [54]. It is intriguing that although both VC and SR spaces possess immune privilege, the vitreal cavity behaved differently to AAV-mediated gene transfer in inducing the systemic immune response against AAV capsid in our experiment. The underlying mechanism is unknown. However, it is possible that the mechanism(s) contributing to the status of immune privilege for the VC versus SR space is (or are) different. One such difference may be due to VC different outflow mechanisms, and anther may be due to its closer proximity to vascular systems than the SR; both of these differences would tend to offer VC more potential for presenting vector capsid antigens to the immune system.

Anatomically, SR space and VC are situated in different compartments of the posterior part of the eye and this may, in part, explain the differences noted in immune response to vector. The SR space is the virtual space between the RPE and photoreceptors and is only present in retinal detachment. The RPE monolayer forms the outer blood-retina-barrier (BRB), separating the vessels of the choriocapillaris from the neural retina, and controls the exchange of molecules and cells between retina and choroid. RPE cells secrete a variety of immune-suppressive and antiinflammatory molecules [55,56]. These cells also express cell membrane-bound molecules, such as FasL (CD95L), which induces apoptosis of CD95^+^ inflammatory cells and contribute to ocular immune privilege [57,58]. More importantly, the SR space is thought to be devoid of bone marrow-derived cells, whereas ACAID is induced by bone-marrow-derived antigen presenting cells (APC) residing in the anterior chamber, which carry an antigen-specific signal via the circulation to the spleen [59]. In contrast, it is still not known how the signals that generate immune deviation following antigen injection into SR space escape the eye.

The vitreous cavity is the most posterior chamber of the eye, situated between the lens and the retina, and is occupied by the gelatinous vitreous body. Sonoda et al. [46] reported that there was an antigen-specific immune deviation when introduced into VC, and named it the vitreous cavity associated immune deviation (VCAID), which shares common mechanisms with ACAID. Interestingly these authors found that bone marrow-derived “hyalocytes” in the vitreous are actually F4/80+, and may serve as APCs responsible for mediating this VCAID. Thus VCAID is more similar to ACAID than the immune deviations induced by antigens present in the SR space. This difference might also contribute to the difference in immune response to AAV vectors when delivered into these two different compartments in our experiments. Further studies are needed to better appreciate these mechanisms and advance our understanding of the pathogenesis of many other ocular diseases of immunological origin.

The results of our present study are consistent with that of Bennett et al. [38] and Anand et al. [39] in that both nonhuman primate and murine RPE and photoreceptor cells can be transduced efficiently by AAV vectors in spite of the presence of circulating antibodies to AAV capsid. However, we did not see detectable antibody against AAV capsid following a single subretinal injection of AAV vector, contrary to that observed by Anand et al. [39, 60]. This difference may result from different injection methods and reagents used, or perhaps to escaped antigen at the injection site following subretinal treatment where there is transient disruption of outer BRB formed by RPE cells since it has been shown that breakdown of RPE barrier function comprises the status of ocular immune privilege [47].

In summary, the findings reported here have important clinical implications in designing gene therapy protocols aimed both at targeting different retinal cell types residing in different ocular compartments and at treating partner eyes sequentially. It may be possible to readminister AAV vectors into the subretinal space to target photoreceptors as well as the RPE without compromising the efficacy of repeated gene transfers. However, for gene transfer targeting the inner retina via the vitreous, other strategies to circumvent immune responses may be required. Such strategies include using different AAV serotypes (see recent review [61]), transient immune suppression, and induction of immune tolerance (see review in [15] and references therein). It should be pointed out that these studies were performed in experimental animals and cannot necessarily be directly extrapolated to humans who comprise a far more immunologically heterogeneous population than any animal research species. It should also be pointed out that many factors contributing to the immune privilege of subretinal space, such as the presence of blood-retinal-barriers, could be compromised under pathological conditions in humans, including diabetic retinopathy, the choriodal neovascularization seen in age-related macular degeneration, or ocular autoimmune diseases. Under such conditions, immune responses to ocular AAV-mediated gene transfer may also be different, and further studies are needed to address these issues.
